# Undifferentiated Pancreatic Carcinoma With Osteoclast-Like Giant Cells and Associated Ductal Adenocarcinoma With Focal Signet-Ring Features

**DOI:** 10.7759/cureus.14988

**Published:** 2021-05-12

**Authors:** Oluwaseyi Olayinka, Gagandeep Kaur, Gunjan Gupta

**Affiliations:** 1 Pathology and Laboratory Medicine, Danbury Hospital, Danbury, USA

**Keywords:** carcinoma pancreas, undifferentiated carcinoma, osteoclast-like giant cells, case report, ductal adenocarcinoma variant

## Abstract

Undifferentiated pancreatic carcinoma with osteoclast-like giant cells (UPC-OGC) is a unique and rare tumor characterized by the presence of highly atypical carcinoma cells and non-neoplastic osteoclast-like giant cells. The histogenesis of this tumor is unclear and data on its prognosis remain controversial. Some data show poor clinical outcomes in affected patients while other more recent studies report a better outcome especially for cases with pure UPC-OGC. There are currently no established reliable management guidelines for UPC-OGC partly because of its rarity and presence of conflicting data in the literature. Hence the need for continued reporting and further research on this neoplasm. We report an incidental finding of UPC-OGC with associated ductal adenocarcinoma and focal signet ring features in an elderly male patient who presented with symptoms of urinary tract infection (UTI).

## Introduction

Undifferentiated pancreatic carcinoma with osteoclast-like giant cells (UPC-OGC) is an extremely rare neoplasm characterized by the presence of three populations of cells: osteoclast-like giant cells, histiocyte-like “sarcomatoid” carcinoma cells, and pleomorphic giant carcinoma cells. The tumor comprises <1% of non-endocrine tumors of the pancreas with the majority of cases occurring between age 60 and 70 years [1,2]. Little is known about the histogenesis of UPC-OGC and data on its prognosis has been controversial. More studies are therefore needed to further characterize and correctly classify the tumor which has been reported to coexist with conventional pancreatic ductal adenocarcinoma, a fatal neoplasm with an overall five-year survival of less than 10% [3].

## Case presentation

We report the case of an elderly male patient with no significant past medical history who was initially hospitalized for bacteremia secondary to urinary tract infection (UTI). During his hospitalization, a retroperitoneal ultrasound showed bilateral renal cysts including a right complex cyst. Further evaluation of the cysts via MRI revealed a large heterogeneous irregularly enhancing mass in the region of the distal pancreatic body measuring approximately 7.2 x 7.1 x 5.9 cm in size which abutted the posterior, medial and superior margin of the stomach, lateral aspect of the celiac axis, and appeared to arise from the pancreas where there was distal pancreatic tail atrophy and ductal dilation. The patient underwent an upper endoscopic ultrasound which identified an oval mass in the pancreatic body. The mass measured 6.8 cm by 4.8 cm in maximal cross-sectional diameter and was isoechoic with several cystic foci measuring up to 1.2 cm. The endosonographic borders were well-defined. Fine needle aspiration (FNA) for cytology and core needle biopsy were performed.

Cytologic examination of the aspirate was positive for malignant cells. Core needle biopsy showed a poorly differentiated malignant neoplasm with giant cells and sarcomatoid areas with tumor cells displaying bizarre cytology and no definable differentiation. Many osteoclast-like giant cells with clusters of 20-40 bland monomorphic nuclei were also seen. Immunohistochemistry revealed the sarcomatoid areas to be negative for cytokeratin (CK) 7, CK 20, CK AE1/AE3, S100, and desmin. CK CAM 5.2 was, however, focally and weakly positive in the sarcomatoid areas. CD68 distinctly highlighted osteoclast-like giant cells. Based on weak, focal staining for CAM 5.2, an undifferentiated carcinoma with osteoclast-like giant cells was favored over a sarcoma with osteoclast-like giant cells. The patient underwent further imaging for pancreatic cancer staging. CT of the chest, abdomen, and pelvis showed no evidence of adenopathy or distant metastasis. The patient subsequently had a distal pancreatectomy and splenectomy with abdominal lymphadenectomy. The specimens were sent for pathologic examination.

**Figure 1:**
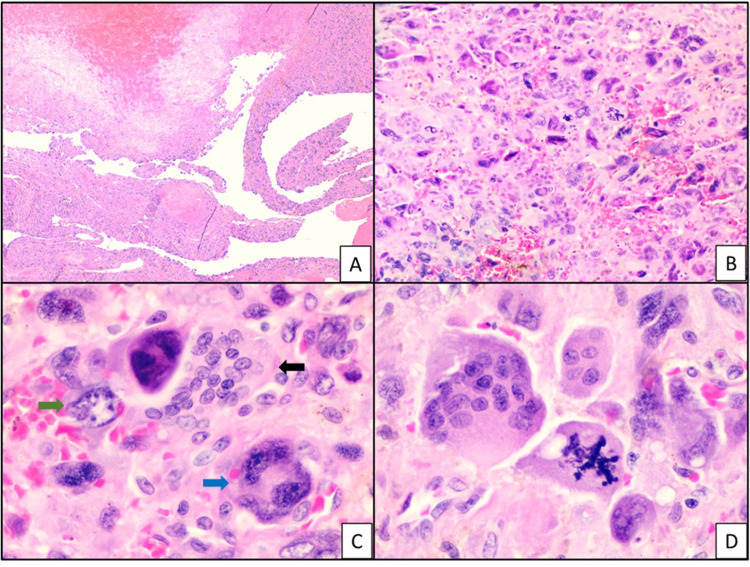
Hematoxylin–eosin showing (a) cystic areas with hemorrhage and necrosis within the tumor, 2X (b, c) undifferentiated component with three distinct cell populations including histiocyte-like sarcomatoid cells (green arrow), giant pleomorphic carcinoma cells (blue arrow), and osteoclast-like giant cells (black arrow), 10X, 40X (d) giant pleomorphic carcinoma cells with atypical mitosis, 40X.

Gross examination of the pancreas showed a 5.0 cm partially cystic, partially solid, tan-red tumor mass attached to the surface of the distal pancreas. Upon sectioning, the solid component revealed areas of hemorrhage and necrosis. The tumor mass was located 2.0 cm from the pancreatic parenchymal surgical margin and did not grossly appear to invade the splenic vein or splenic artery. The remainder of the resected pancreas was tan-white and grey with no other gross lesions. The spleen was unremarkable. Microscopic examination showed a malignant neoplasm with two components. One was an undifferentiated component comprised of complex cellular and hemorrhagic nodules with three distinct cell populations including histiocyte-like and giant pleomorphic carcinoma cells with osteoclast-like giant cells (Figure 1). 

**Figure 2:**
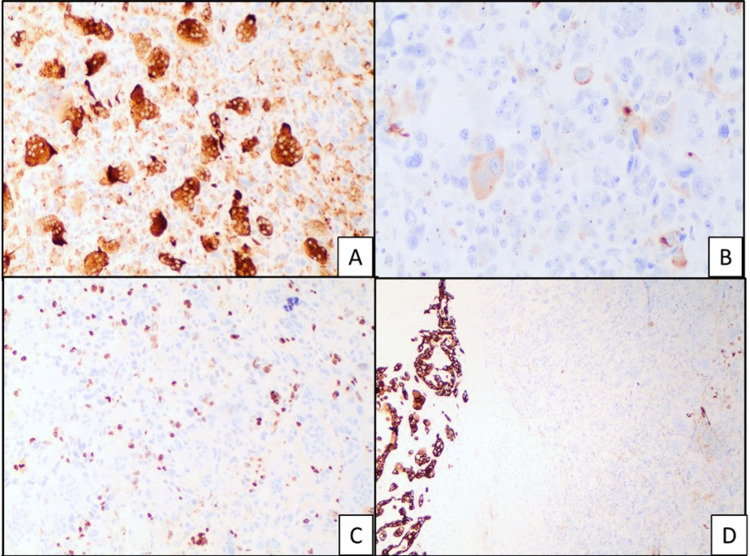
Immunohistochemical stain showing (a) osteoclast-like giant cells and histiocyte-like cells are positive for CD68, 20X (b) histiocyte-like carcinoma cells and pleomorphic giant carcinoma cells are focally positive for CAM 5.2, 20X (c) high (Ki-67) proliferation index in histiocyte-like carcinoma cells and pleomorphic giant carcinoma cells, 10X (d) undifferentiated component with focal staining for CAM 5.2 (right) and ductal adenocarcinoma component with strong staining for CAM 5.2 (left), 2X.

**Figure 3:**
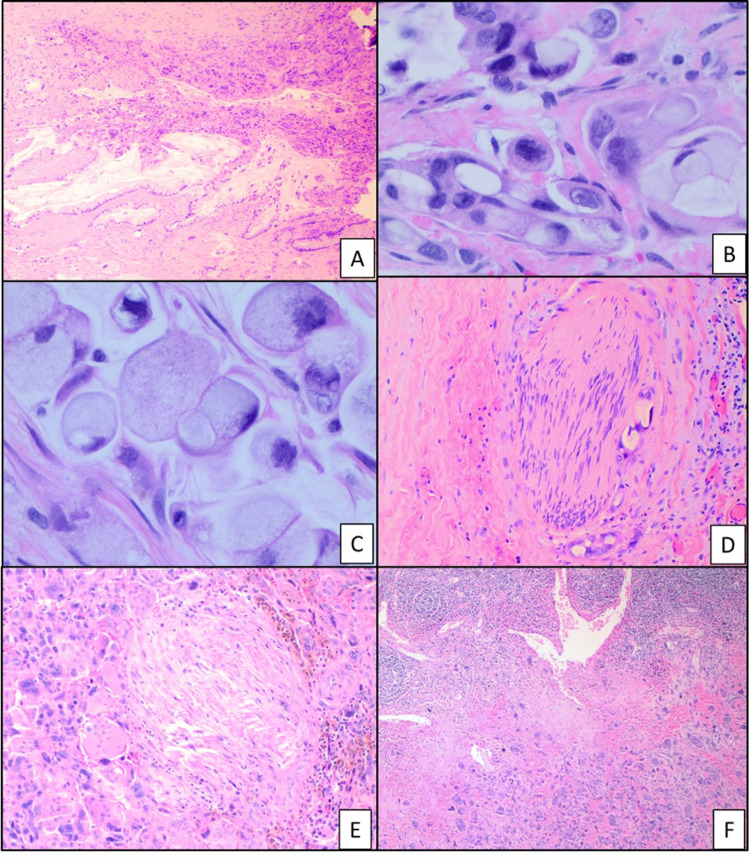
Hematoxylin–eosin showing (a) undifferentiated component with ductal adenocarcinoma, 4X (b, c) ductal adenocarcinoma and signet ring cells, 40X. (d, e) perineural invasion, 20X (f) peripancreatic lymph node with metastatic carcinoma, 4X.

Immunohistochemical analysis showed that the histiocyte-like carcinoma cells, as well as the pleomorphic giant carcinoma cells, are focally positive for CAM 5.2. These cells were negative for CK AE1/AE3 stain and showed a high (Ki-67) proliferation index. The osteoclast-like giant cells and histiocyte-like cells were positive for CD68 while the pleomorphic carcinoma cells were negative for this stain (Figure 2). The osteoclast-like giant cells had a low Ki-67 index and p53 stained rare scattered cells. A second minor component was also seen which showed ductal adenocarcinoma highlighted by AE1/AE3 and CAM 5.2 stains with a small focus of signet ring cells (Figures 2-3). Lymphovascular and perineural invasion was present and the tumor cells were present within the peripancreatic soft tissues. The proximal pancreatic parenchymal margin was negative for neoplasia. One of 25 peripancreatic lymph nodes was positive for metastatic carcinoma (Figure 3).

The two excised hepatic artery lymph nodes were negative for tumor. The spleen showed no significant histopathologic changes. A final diagnosis of undifferentiated pancreatic carcinoma with osteoclast-like giant cells and associated moderately differentiated pancreatic ductal adenocarcinoma with focal signet ring features was made. Three months post-surgery, the patient is healing well and currently receiving adjuvant chemotherapy with gemcitabine and capecitabine with minimal side effects.

## Discussion

UPC-OGC is a very rare neoplasm comprising <1% of non-endocrine pancreatic tumors [1]. Patients commonly present in the sixth to seventh decade of life and the disease shows a slight female predominance. Although believed to originate from pancreatic ductal epithelial cells and classified by the World Health Organization as a histologic subtype of pancreatic ductal adenocarcinoma, the exact histogenesis of UPC-OGC remains unclear [4]. Patients with UPC-OGC can easily be misdiagnosed with intestinal or pancreatic inflammatory diseases as the symptoms are often non-specific [2]. Reported clinical manifestations include abdominal pain, anorexia, fatigue, weight loss, jaundice, and a palpable mass [1]. In our case study, the patient was initially asymptomatic but later admitted to having fatigue while been worked up for multiple cysts. Serum CA 19-9 levels may be elevated in patients with UPC-OGC. In one study, serum CA 19-9 was elevated in 73% of patients [5]. Our patient had a normal serum CA 19-9. UPC-OGC is a relatively large tumor and is more often seen in the body/tail of the pancreas when compared to pancreatic ductal adenocarcinomas without osteoclast-like cells [5,6]. On imaging, the tumor commonly appears as a heterogeneous mass with distinct hyper- and hypoechoic regions unlike typical adenocarcinoma of the pancreas which is uniformly hypoechoic [6]. The image usually shows a solid cyst with a clear boundary and there is often hemorrhage and necrosis in the cyst [2].

Diagnosis of UPC-OGC requires examination of tissue samples. Preoperative biopsy often demonstrates malignancy with about a third of patients receiving a diagnosis of UPC-OGC [5]. Our patient had an FNA which demonstrated malignant cells followed by a core needle biopsy that favored a diagnosis of UPC-OGC. The pathologic features of UPC-OGC are distinctive. The tumor displays a nodular/pushing-border growth pattern and often shows prominent intraductal/intracystic polypoid growth. They tend to be hemorrhagic and often show cystic degeneration. On microscopic examination, the tumor characteristically shows osteoclast-like giant cells containing over 20 relatively uniform, bland nuclei existing in a background of histiocyte-like “sarcomatoid” carcinoma cells and pleomorphic giant carcinoma cells with large irregular hyperchromatic nuclei. The osteoclast-like giant cells which are positive for CD68 with a low Ki-67 index are non-neoplastic and believed to have been recruited by the tumor’s sarcomatoid component via mechanisms still unknown. Most of the histiocyte-like carcinoma cells have also been shown to express CD68 with focal, mostly weak, staining for AE1/AE3. Both the histiocyte-like and pleomorphic giant carcinoma cells have a high proliferation rate with Ki-67 stain with the latter cell type also demonstrating focal, weak, staining for AE1/AE3 [5]. In our study, both cell types were negative for AE1/AE3 but focally and weakly positive for CAM 5.2. Histiocyte-like and pleomorphic giant carcinoma cells have a high expression of p53 while the osteoclast-like cells are negative for p53. Other known molecular characteristics of UPC-OGC include activating mutations in the oncogene KRAS and inactivating mutations in the tumor suppressor genes CDKN2A, and SMAD4 similar to genetic alterations seen in conventional ductal adenocarcinoma [7].

Although present in our case, UPC-OGC is less likely than ductal adenocarcinoma to show perineural invasion and lymph node metastasis. Some UPC-OGCs are associated with conventional ductal adenocarcinoma while others arise in a mucinous cystic neoplasm (MCN) or intraductal papillary mucinous neoplasm (IPMN). Our case was associated with conventional ductal adenocarcinoma and a focus of signet-ring cells was identified. It is unclear if the presence of other tumor types or the presence of signet-ring cells changes the clinical behavior of UPC-OGC although more recent studies show better survival for patients with pure UPC-OGC [5,7,8]. In the study by Muraki et al., the five-year survival of patients with no ductal component was 100%. No significant difference was documented between cases with and without intraductal/intracystic growth, MCN/IPMN, or the presence of an osteoid component [5]. The overall survival of all UPC-OGC was found to be significantly better than that of pancreatic ductal adenocarcinoma. This is contrary to a previous report that UPC-OGC is an aggressive tumor with a poor prognosis [9]. In one study, the authors report specific characteristics of short-term survivors of UPC-OGC (those who succumbed within one year following surgical resection) including older age, male gender, smaller tumor size, lymph node metastasis, and presence of a ductal adenocarcinoma component, as well as pleomorphic giant cell carcinoma [10]. 

There are currently no established reliable management guidelines for UPC-OGC [11]. Operative intervention is often used as first-line treatment in surgically resected tumors. Some patients undergo chemotherapy with drugs such as gemcitabine and/or radiation therapy though the role of both therapies either as adjuvant or neoadjuvant agents has not been clearly established [2,12,13]. Our patient had surgical resection and is currently receiving gemcitabine and capecitabine as adjuvant chemotherapy.

## Conclusions

This case report highlights the incidental finding of UPC-OGC in an elderly male patient who presented with symptoms of UTI. The histogenesis of the tumor remains unclear though recent clinicopathologic and molecular studies suggest an epithelial origin. Recent analysis also shows a better clinical course especially in patients with pure UPC-OGC. However, further research on this rare entity is needed to confirm or refute these data. The goal is to correctly characterize the tumor in order to offer appropriate care and prolong survival.
